# Effect of nanohydroxyapatite paste after different pretreatment techniques on remineralization and color change of white spot lesions in children: a randomized control study

**DOI:** 10.1186/s12903-025-07365-5

**Published:** 2026-01-13

**Authors:** Maryam Mohamed El Mansy, Reham Sayed Saleh, Mohamed Farouk Rashed, Ahmed Kamal El-Motayam

**Affiliations:** 1 Researcher of Pediatric Dentistry, Orthodontics and Pediatric Dentistry Department, Oral and Dental Research Institute, National Research Centre, 33 El Buhouth St, Dokki, Giza Governorate, 12622 Egypt; 2 Associate Professor, Restorative and Dental Material Department, Oral and Dental Research Institute, National Research Centre, 33 El Buhouth St, Dokki, Giza Governorate, 12622 Egypt; 3 Associate Professor, Department of Pediatric Dentistry and Dental Public Health, Faculty of Dentistry, Cairo University, Al Saraya, Old Cairo, Cairo, 12613 Egypt

**Keywords:** White spots, Nanohydroxyapatite, Microbrasion, NaOCl, Diagnodent, Vita easy shade

## Abstract

**Background:**

White spot lesions (WSLs) are initial caries caused by early demineralization. Nanohydroxyapatite (NHA) paste is one of the most recent non-fluoridated remineralizing agents; however, few clinical studies have reported its remineralizing effect on such lesions. The aim of this study was to compare and evaluate the remineralization and color change of WSL after NHA alone was used versus NHA pretreatment with either microabrasion or sodium hypochlorite (NaOCl) and no treatment as a negative control.

**Methods:**

Twenty children aged 10 to 14 years with 40 teeth affected with WSL; were randomly assigned to 4 groups (10 teeth in each group); group I: pretreatment with microabrasion followed by NHA paste, group II: pretreatment with 5.25% NaOCl, followed by NHA paste, group III: NHA paste alone, and group IV: negative control with no treatment applied. The remineralization ability was assessed via diagnodent device and color changes were assessed via a Vita easy shade device before and after treatment; and one, two and six weeks after treatment.

**Results:**

For the change in Diagnodent readings, there was a significant difference between groups (I, II, III) at different intervals, which as significantly greater than that of group IV at a p value < 0.001. However, for Group IV, the change was not statistically significant (*p* = 0.332). The color changes measured after 2 and 6 weeks were significantly greater in groups (I), (II) and (III) than group IV (*p* < 0.001).

**Conclusions:**

NHA could be a good non fluoridated option to remineralize and improve the color of WSL at different treatment intervals. Pretreatment with microabrasion and NaOCl enhances its remineralization and color improvement properties.

**Trial registration:**

This study was retrospectively registered on ClinicalTrials.gov at 6/2/2025, with ID: NCT06834464.

**Supplementary Information:**

The online version contains supplementary material available at 10.1186/s12903-025-07365-5.

## Background

 A White spot lesion (WSL) is an initial non cavitated lesion due to the early demineralization stage of the caries process. It can be remineralized to some extent by saliva, although this is a slow process. Such lesions are commonly associated with fixed orthodontic treatment. It causes aesthetic impairment, especially in children and young individuals because of its obviously opaque white appearance, and if left untreated, it might lead to an advanced and cavitated carious lesion. In addition, enamel demineralization may occur during the eruptive phase as in fluorosis and Molar Incisor Hypomineralization [1].

The prevalence of white spot lesions has been reported to range from 5% in children without orthodontic treatment to 97% in orthodontically treated individuals, and the labial surfaces of maxillary incisors are most commonly affected [2].

Several conservative remineralizing therapies have been used in the management of carious non cavitated lesions. However, fluoride is still the traditional preventive agent that has been widely used in dentistry due to its potent cariostatic properties. Nowadays, it is noticed that the prevalence of dental caries in many in countries is declined because of the widespread use of fluoridated the toothpastes [3].

On the other hand, it has been documented that exposure to chronic low‑level fluoride can present several problems of normal individuals. In addition, it is noticed that the prevalence of dental fluorosis is noticeably increasing in nonfluoridated areas and to a lesser extent in optimally fluoridated areas. Moreover, fluoride does not remineralize the deepest part of the lesion; consequently, it does not improve aesthetics [4].

To improve enamel remineralization, a variety of novel therapeutic agents, including non-fluoridated items such as bioactive glasses and casein phosphopeptide amorphous calcium phosphate (CPP-ACP) have been lunched in the market [5].

Recently, nanotechnology has attracted a great attention in different medical fields. Over the years, several studies on NHA as a biomimetic material have recommended its use for the reconstruction of tooth enamel that lost its mineral [6, 7].

Nanohydroxyapatite has been found to possess similar morphology, structure, and crystallinity as a.

biological apatite. In addition, it is more biocompatible than larger hydroxyappatite (HA) materials. Moreover, it can significantly increase the degree of remineralization by facilitating more ion diffusion into the center of the demineralized zone [8].

The pretreatment approach has gained popularity in the management of WSL. Rapid remineralization of the enamel surface with salivary high concentration of fluoride, renders ions passage into the deeper and more affected layers. Therefore, immediate application of a high concentration of fluoride is not advised. Some studies have commended the application of different pretreatment protocols to remove this layer to allow further passage of remineralizing particles into the deeper layer [9, 10].

Microabrasion is a reliable and conservative treatment option that makes the enamel surface smoother and free of surface irregularities. Additionally, it produces a more regular and shiny surface, and removes opaque and brown stains [11].

The advancement in remineralizing therapies and new conservative approaches to manage noncarious lesions have cached the interest towards the detection and monitoring such precavitated lesions. One of these available noninvasive methods is Diagnodent. DIAGNOdent^®^ (KaVo) uses laser fluorescence (LF) to measure early demineralization [12, 13].

Visual assessment of tooth color is considered highly subjective and inconsistent because of several variables can be either external variables such as light conditions, or internal variables such as experience, age and fatigue of the human eye. Hence, the vita easyshade spectrophotometer is used to detect color changes because of its precision, numerical expression of color, and lack of subjective bias [14, 15].

To our knowledge, no clinical study has been conducted in the literature comparing the effects of NHA with or without pretreatment modalities with those conventional oral hygiene measures alone for conservative management of WSL on anterior teeth.

The specific objectives of this study were to compare and evaluate the remineralization and color change of WSL after the use of NHA alone versus pretreatment with microabrasion followed by NHA, or pretreatment with NaOCl followed by NHA and no treatment as a negative control.

For the treatment of white spot lesions; the null hypothesis assumes that none of the applied pretreatment protocols either microabrasion or NaOCl followed by NHA, can remineralize or improve the color of the white spot lesion in comparison to NHA used without pretreatment.

## Materials and methods

### Study design

**Fig. 1:**
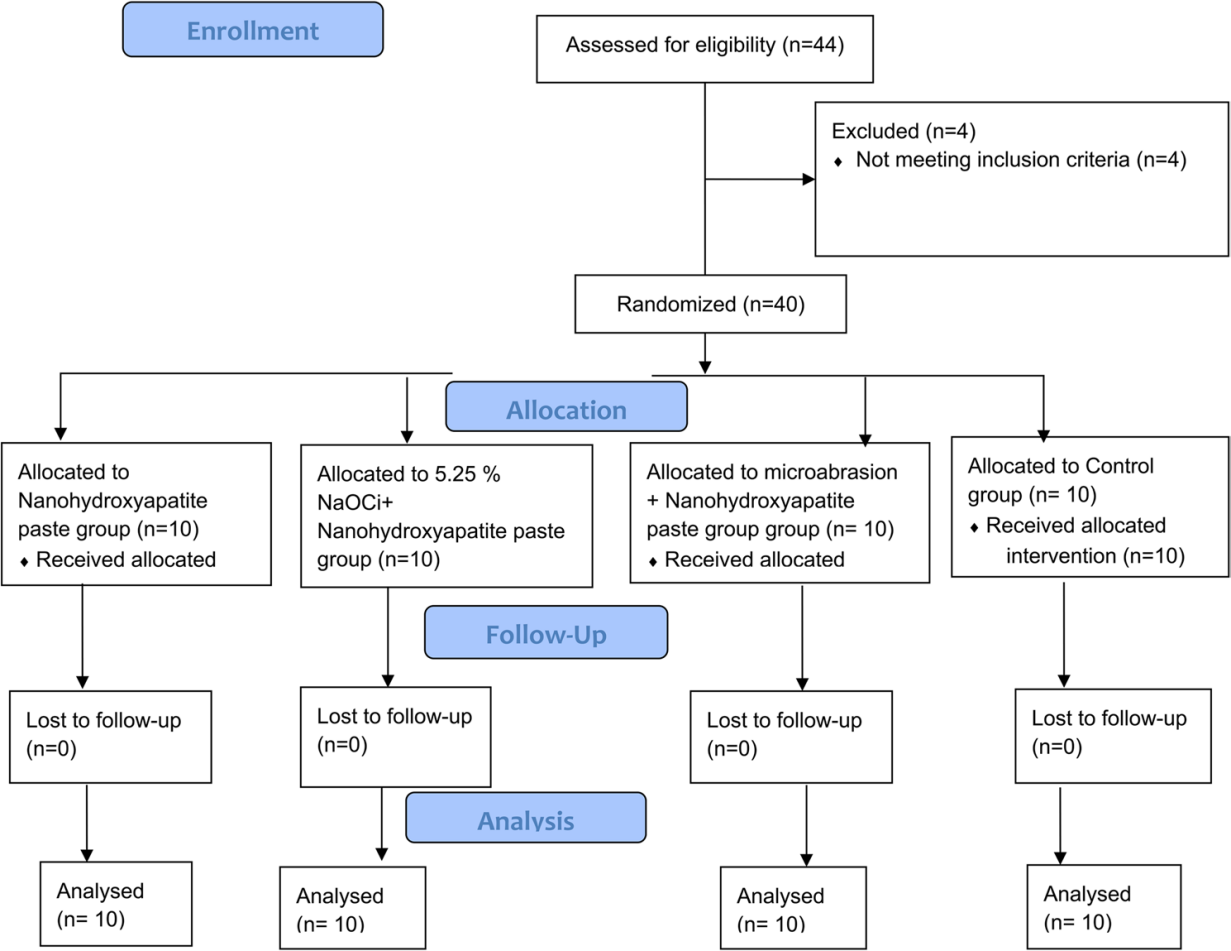
Participants flow diagram

The study was planned as a triple blinded randomized controlled trial (RCT). This study was adhered to the Consolidated Standards of Reporting Trials (CONSORT) guidelines at 2010 for the designing and reporting of clinical trials [16]. In this study, twenty-two participants were enrolled. Twenty patients with forty anterior permanent teeth diagnosed with non-cavitated white spot lesions were allocated randomly to our study, received the planned intervention, and were subsequently assessed for various outcomes at various stages of the study, as illustrated in the CONSORT 2010 flowchart presented in Fig. 1.

### Ethical approval and clinical trial registration

The Medical Research Ethical Committee at the National Research Centre, Egypt, approved the study with number: 0343 on November 26, 2024, in consistent with the ethical standards put down in the 1964 declaration of Helsinki and the amendments that set later on [17]. This study was retrospectively registered on ClinicalTrials.gov at 6/2/2025, with ID: NCT06834464.

### Participants

Twenty-two children of both sexes aged 10–14 years were chosen among from those referred to the Pedodontic Department throughout the study period. The children’s eligibility for this study was assessed by the principal investigator where two patients were rejected because of presence of hypoplastic teeth. Twenty children were eligible for our study on the basis of the following eligibility criteria:

### The inclusion criteria

The inclusion criteria were healthy patients with at least 1 WSL on the labial surface of one or more permanent upper or lower anterior teeth, who had not used any fluoride regimens, and who were 10–14 years of age.

### The exclusion criteria were as follows

Patients with any chronic medical problem that could impact the study results, or who had received or were applying fluoride treatment for white spot lesions right now. Teeth with cavitated or hypoplastic lesions were also excluded [10].

### Setting and location

Children recruitment was carried out from the outpatient dental clinics at the National Research Centre, Egypt. Children were screened for diagnosis between November and December 2024. The follow-up period ended in February 2025.

### Sample size calculation

According to a previous study by Mohammed et al. (2021) [18], the mean shade varied between 20.75 ± 2.83, 20.0 ± 3.18 and 21.35 ± 2.98 in different groups. Using the G power statistical power analysis program (version 3.1.9.4) for sample size determination [19], a total sample size (*n* = 40; subdivided into 10 teeth in each group) was sufficient to detect a large effect size (f) = 0.58, with an actual power (1-β error) of 0.8 (80%) and a significance level (α error) of 0.05 (5%) for two-sided hypothesis testing.

### Intervention

#### Informed consent

Before beginning the intervention, parents signed a written informed consent form after being briefed on the procedures in simple language. The child also gave verbal agreement preoperatively.

#### Randomization

The participating children were allocated to the 4 study groups at a 1:1 allocation ratio, and randomization was carried out via the website www.random.org, which on 15 November 2024. Thus, the children were assigned to 4 groups; group I: pretreatment with microabrasion followed by NHA paste, group II: pretreatment with 5.25% NaOCl, followed by NHA paste, group III: NHA paste alone, and group IV: negative control with no treatment applied.

### Allocation concealment

The random sequence in on the website (random.org) was generated by RS. While the sequence of allocation was kept with AK to ensure total concealment. Operator MF and MM selected the patients and AK released the allocation for each patient at the treatment appointment. MF and MM provided patient treatment.

#### Blinding

In this study, a triple-blinded approach was followed including examiners, assessors, treating clinicians, children and statisticians. Outcomes were evaluated by a well-trained researcher who didn’t know the performed intervention in each case.

#### Clinical procedures

I. Diagnosis of white spot lesions:

A) Visual examination:
  Clinically, the lesion appears opaque white, under dry conditions with good illumination [20].
  First, air dryness of the tooth surface for at least 5 seconds was done after cleaning with pumice. Then tooth was examined with the help of light and a mirror in order to correctly diagnose the WSL [21].

While Vita easy shade as an adjunctive tool wasn’t used for diagnosis of the WSLs but it was used to record the base line color of these lesions.

B) Laser fluorescence device “DIAGNOdent:
  Laser fluorescence device “DIAGNOdent® (KaVo)” was used for demineralization assessment.
  A Type B tip, designed for flat surfaces such as front tooth labials, was used for all measurements and before each measurement; caeramic standard was used for calibration according to the manufacturer’s instructions [22].
  After calibration, the DIAGNOdent device was implanted by maintaining the tip close to the tooth surface and tilting it around the measuring region to collect fluorescence from all angles. In our study, all teeth with a moment value above 7 on the digital display (a DIAGNOdent score between 3 and 7 indicates normal enamel) indicated subsurface demineralization of the tooth surface [23].
  All measurements were recorded by two blinded external investigators.

I. Grouping of samples:
  Group 1: Pretreatment with microabrasion (Oplasture) for 60 seconds after which NHA paste (Sangi Apagard Royal NHA paste) was applied for 5 minutes.
  Group 2: Pretreatment was performed with 5.25% NaOCl for 20 seconds after which NHA paste (Sangi Apagard Royal NHA paste) was applied for 5 minutes.
  For Group 3: NHA paste (Sangi Apagard Royal NHA paste) was applied for 5 minutes.
  In Group 4: no treatment was applied (oral hygiene instructions were given only) (negative control group).

I. Treatment modalities:

i. Preoperative preparation:

Before the studied materials were applied, the operator asked all the children involved in this study to brush their teeth without toothpaste under the parents’ supervision.

The labial surfaces of the teeth in the study group were cleaned with water slurry of pumice and rubber prophy cups. After cleaning and drying with a cotton roll, a cheek retractor and saliva ejector were inserted to permit precise application of the tested material without dissolution by saliva.

ii. Grouping of subjects:

- Group 1: Oplasture microabrasion + NHA paste group:

i. Microabrasion:

The initial step of microabrasion was isolation of the teeth from the lips and gum. In addition, Opaldam® (Ultradent Products, UT, USA) was applied to protect the gingiva.

The second step was the application of Opalustre® (Ultradent Products, UT, USA) on the surface of the teeth for 30 seconds; then the material was rubbed via a hybrid bristled cup; OpalCups (Ultradent Products, UT, USA) for another 30 seconds followed by copious amount of irrigation then drying [24].

ii. A thin layer of NHA paste was placed with a plastic applicator on the surface of the affected teeth for 5 minutes.

- Group 2: 5.25% NaOCl + NHA paste group:

Pretreatment of the affected teeth was performed via applying 5.25% NaOCl (Adam dent, Egypt) by a cotton swab on the surface of the teeth for 20 seconds. The teeth were washed for 10 seconds with a water syringe then dried with an air syringe for 5 seconds [25].

The NHA paste was applied in the same manner as in group one.

- Group 3: NHA paste group:

The participants in this group were treated by NHA remineralizing paste (Apagard Royal NHA paste, Sangi, Japan).

The NHA paste was applied in the same manner as in group one.

-The children were instructed to stop eating or drinking for one hour after treatment in these groups.

- Group 4: negative control group:

No treatment was applied (oral hygiene instructions were given only by brushing their teeth twice per day with fluoridated toothpaste) (control group).

Oral hygiene instructions including brushing twice daily with fluoridated toothpaste were given equally to all study groups. Every observation period, repetition of oral hygiene instructions was carried out to eliminate any confounding effect on the outcomes.

### Outcome assessment

#### Laser fluorescence device “DIAGNOdent

Assessment of the lesions’ severity was measured via DIAGNOdent laser fluorescence device before the application of the remineralizing paste and another reading was recorded after 10 min of application [22].

All the measurements were performed according to the manufacturer’s instructions in the same manner as those used for the diagnosis of WSLs.

Other recordings were taken at one week, two weeks and six weeks from teeth in each of the study and control groups. Differences between recorded values at each visit were analyzed for statistical analysis [21].

#### Color assessment

A Vita Easyshade spectrophotometer (Vita Zahnfabrik, Bad Säckingen, Germany) was used for color assessment of the WSLs.

Prior to each measurement, the device was calibrated according to the manufacturer’s instructions using a white table supplied by the manufacturer. Evaluation of the tooth color was done by holding the probe tip at a right angle to the labial surface of the tooth. Then values were recorded according to the Commission Internationale de l’Eclairage L* a* and b* (CIELAB) color space system. Where “L” axis stand for the degree of lightness, while “a” values sand for the positions on the red/green (+ a = red, −a = green) axes and “b” value stand for the position on the yellow/blue (+ b = yellow, −b = blue) axes. For each tooth, color assessment was performed at central area of the WSL and on a healthy tooth structure [26].

The assessments were repeated three times to minimize measurement errors, then the mean value was recorded for each area. At each follow‑up appointment the color assessment was repeated under the same conditions.

The color difference of the WSL between different intervals was calculated via the following formula:

$$\triangle E_{00}\left({L^\ast}_{1,}{a^\ast}_{1,}{b^\ast}_{1;}{L^\ast}_{2,}{a^\ast}_{2,}{b^\ast}_2\right)=\triangle{E^{12}}_{00}=\triangle E_{00.}$$

The color change of the WSLs was assessed; before treatment, immediately after treatment, one week, two weeks, and six weeks after the onset of treatment on each tooth in each of the study groups and the control group via a Vita easy shade device [27].

Both color and remineralization measurements were recorded by two blinded external investigators.

**Fig. 2:**
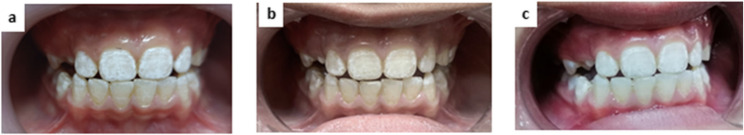
Shows color change in upper central incisors for a case in microabrasion +NHA group; (**a**) preoperatively, (**b**) after 1 week and (**c**) after 6 weeks

**Fig. 3:**
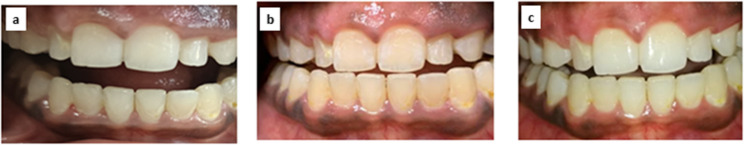
Shows color change in upper central incisors for a case in NaOCl +NHA group; (**a**) preoperatively, (**b**) after 1 week and (**c**) after 6 weeks

**Fig. 4:**
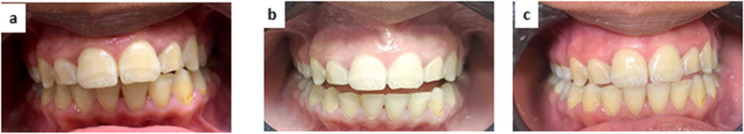
Shows color change in upper central incisors for a case in NHA only group; (**a**) preoperatively, (**b**) after 1 week and (**c**) after 6 weeks

Examples of clinical cases from various groups at different intervals are shown in Figs. 2, 3 and 4.

### Statistical analysis

Numerical data differences are presented as mean with 95% confidence intervals (CIs), median, and interquartile range (IQR) values. Reading differences were analyzed for normality by viewing the distribution and using Shapiro-Wilk’s test and were found to be non-parametric. Intergroup comparisons were analyzed using Kruskal-Wallis and Dunn’s tests. Intragroup comparisons were analyzed using Freidman’s and Nemenyi’s tests. P-values were adjusted for multiple comparisons using the false discovery rate (FDR) method. Effect sizes were calculated and interpreted based on Maciej Tomczak and Ewa Tomczak [28]. Spearman’s rank-order correlation coefficient was used C for correlation analysis.

Statistical analysis was performed with R statistical analysis software version 4.4.2 for Windows [29].

## Results

A total of 20 patients (13 females and 7 males); mean age 12.4 ± 1.1 years completed the study.

**Fig. 5:**
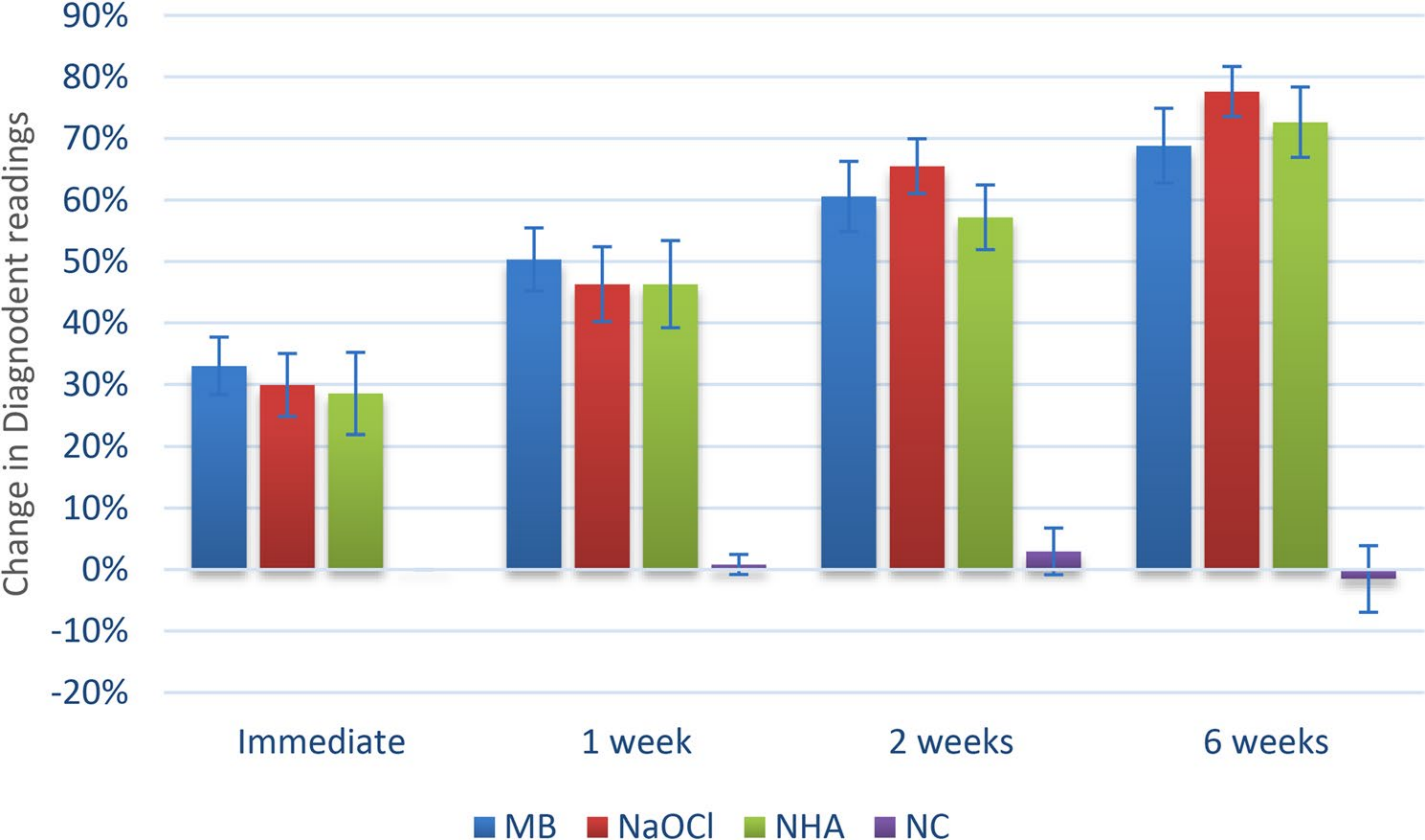
Bar chart showing mean and confidence intervals (error bars) for the change in Diagnodent readings

For the change in Diagnodent readings, as shown in Table 1 and Fig. 5, there was a significant difference between the tested groups at different intervals, with the changes measured in groups I (micro Ab + NHA), II (NaOCl + NHA), and III (NHA only) being significantly greater than those in group IV (no treatment) with a p value < 0.001. Within groups (I) to (III), there was a continuous significant increase in the values measured over time (*p* < 0.001). However, for Group IV (no treatment), the change was not statistically significant (*p* = 0.332).

**Table 1:** Inter and intragroup comparisons of diagnodent readings differences

Interval	Measur-ement changes	Diagnodent reading differences (%)				*p*-value	Effect size	
		Group I (Micro Ab+ NHA)	Group II (NaOCl + NHA)	Group III (NHA only)	Group I (no treatment)		Eta2 [H] (95% CI)	Magni tude
Immediate	Mean (95% CI)	33.02 (28.34:37.70)^Ad^	29.92 (24.80:35.04)^Ad^	28.57 (21.92:35.22)^Ad^	0.00 (0.00:0.00)^Ba^	<0.001*	0.440 (0.200:0.690)	Large
	Median (IQR)	34.52 (12.05)^Ad^	30.00 (7.81)^Ad^	29.17 (19.95)^Ad^	0.00 (0.00)^Ba^			
1 week	Mean (95% CI)	50.34 (45.23:55.46)^Ac^	46.31 (40.22:52.39)^Ac^	46.31 (39.22:53.40)^Ac^	0.83 (0.80:2.47)^Ba^	<0.001*	0.424 (0.190:0.660)	Large
	Median (IQR)	48.53 (9.83)^Ac^	45.44 (10.85)^Ac^	50.00 (19.20)^Ac^	0.00 (0.00)^Ba^			
2 weeks	Mean (95% CI)	60.57 (54.87:66.27)^Ab^	65.49 (61.04:69.95)^Ab^	57.18 (51.92:62.43)^Ab^	2.95 (0.84:6.75)^Ba^	<0.001*	0.466 (0.220:0.700)	Large
	Median (IQR)	60.56 (13.68)^Ab^	65.48 (8.54)^Ab^	58.57 (14.26)^Ab^	0.00 (2.78)^Ba^			
6 weeks	Mean (95% CI)	68.82 (62.74:74.89)^Aa^	77.63 (73.57:81.69)^Aa^	72.64 (66.92:78.35)^Aa^	−1.53 (6.94:3.88)^Ba^	<0.001*	0.462 (0.230:0.690)	Large
	Median (IQR)	69.62 (17.49)^Aa^	80.00 (2.25)^Aa^	73.95 (15.12)^Aa^	0.00 (1.67)^Ba^			
p-value		<0.001*	<0.001*	<0.001*	0.332			
Effect size	Kendall’s W (95% CI)	0.939 (0.900:0.980)	0.972 (0.950:1.000)	0.966 (0.950:0.990)	0.142 (0.050:0.540)			
	Magnitude	Large	Large	Large	Small			

Post hoc test: values with different upper and lowercase superscripts within the same horizontal row and vertical column respectively, are significantly different

*significant (*p*<0.05)

**Fig. 6:**
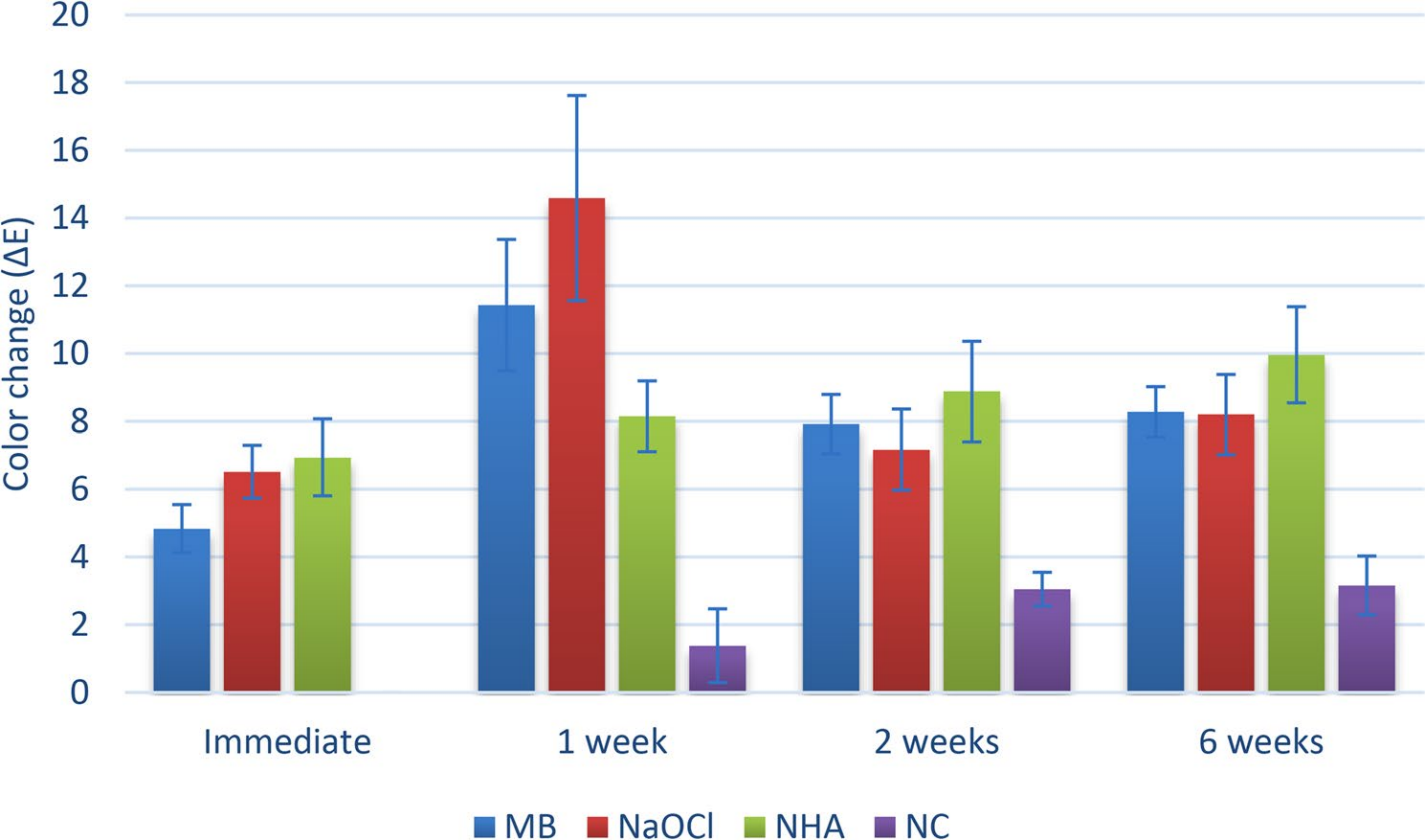
Bar chart showing mean and confidence intervals (error bars) for color change data

For the color change, as shown in Table 2 and Fig. 6, the differences between groups were similarly statistically significant at different intervals (*p* < 0.001). For the color changes measured immediately, in groups II (NaOCl + NHA), and III (NHA only) were significantly greater than in other groups (*p* < 0.001). Additionally, the change measured in group I (Micro Ab + NHA), was significantly greater than that in group IV (no treatment) (*p* < 0.001). At the 1-week change, group II (NaOCl + NHA), had significantly greater values than groups III (NHA only)) and IV (no treatment) (*p* < 0.001). In addition, group I (Micro Ab + NHA) had significantly greater values than did group IV (no treatment) (*p* < 0.001). The changes measured after 2 and 6 weeks, were all significantly greater in groups (I), (II) and (III) than group IV (no treatment) (*p* < 0.001).

**Table 2:** Inter and intragroup comparisons of color change

Interval	Measur-ement change	Color change (ΔE)				*p*-value	Effect size	
		Group I (Micro Ab+ NHA)	Group II (NaOCl + NHA)	Group III (NHA only)	Group I (no treatment)		Eta2 [H] (95% CI)	Magnitude
Imme-diate	Mean (95% CI)	4.84 (4.12:5.55)^Bd^	6.52 (5.74:7.30)^Ac^	6.94 (5.81:8.07)^Ab^	0.00 (0.00:0.00)^Cb^	<0.001*	0.613 (0.380:0.820)	Large
	Median (IQR)	4.68 (1.84)^Bd^	6.29 (1.87)^Ac^	6.52 (2.71)^Ab^	0.00 (0.00)^Cb^			
1 week	Mean (95% CI)	11.43 (9.49:13.37)^ABa^	14.59 (11.57:17.62)^Aa^	8.15 (7.11:9.19)^BCab^	1.38 (0.29:2.47)^Cb^	<0.001*	0.646 (0.440:0.820)	Large
	Median (IQR)	10.62 (4.36)^ABa^	14.47 (3.80)^Aa^	7.69 (1.92)^BCab^	0.78 (2.96)^Cb^			
2 weeks	Mean (95% CI)	7.92 (7.05:8.79)^Ac^	7.17 (5.98:8.36)^Ac^	8.88 (7.40:10.36)^Aab^	3.05 (2.55:3.55)^Ba^	<0.001*	0.460 (0.240:0.700)	Large
	Median (IQR)	7.88 (1.91)^Ac^	6.84 (2.34)^Ac^	8.99 (3.34)^Aab^	3.09 (0.28)^Ba^			
6 weeks	Mean (95% CI)	8.28 (7.54:9.01)^Ab^	8.20 (7.02:9.38)^Ab^	9.96 (8.53:11.38)^Aa^	3.16 (2.30:4.03)^Ba^	<0.001*	0.481 (0.250:0.730)	Large
	Median (IQR)	8.14 (1.06)^Ab^	8.84 (2.68)^Ab^	10.44 (2.68)^Aa^	2.71 (1.06)^Ba^			
p-value		<0.001*	<0.001*	0.033*	<0.001*			
Effect size	Kendall’s W (95% CI)	0.755 (0.650:0.890)	0.708 (0.650:0.870)	0.292 (0.080:0.740)	0.711 (0.610:0.900)			
	Magnitude	Large	Large	Large	Large			

Post hoc test: values with *different upper and lowercase superscripts* within the *same horizontal row and vertical column*, respectively, are significantly different

*significant (*p*<0.05)

Within the all groups, there was a significant difference between the color change values measured at different intervals (*p* < 0.001). For group I (Micro Ab + NHA), all comparisons were statistically significant, with the greatest change measured after one week, followed by 6, 2 weeks, and the immediate change (*p* < 0.001). For group II (NaOCl + NHA), the change measured after one week was significantly greater than that of the other intervals (*p* < 0.001). Additionally, the change measured after 6 weeks was significantly greater than that measured immediately and after 2 weeks (*p* < 0.001). For Group III (NHA only), the change measured after 6 weeks was significantly greater than that measured immediately (*p* < 0.001). For group IV (no treatment), the changes measured after 2 and 6 weeks were significantly greater than those measured at other intervals (*p* < 0.001).

**Fig. 7:**
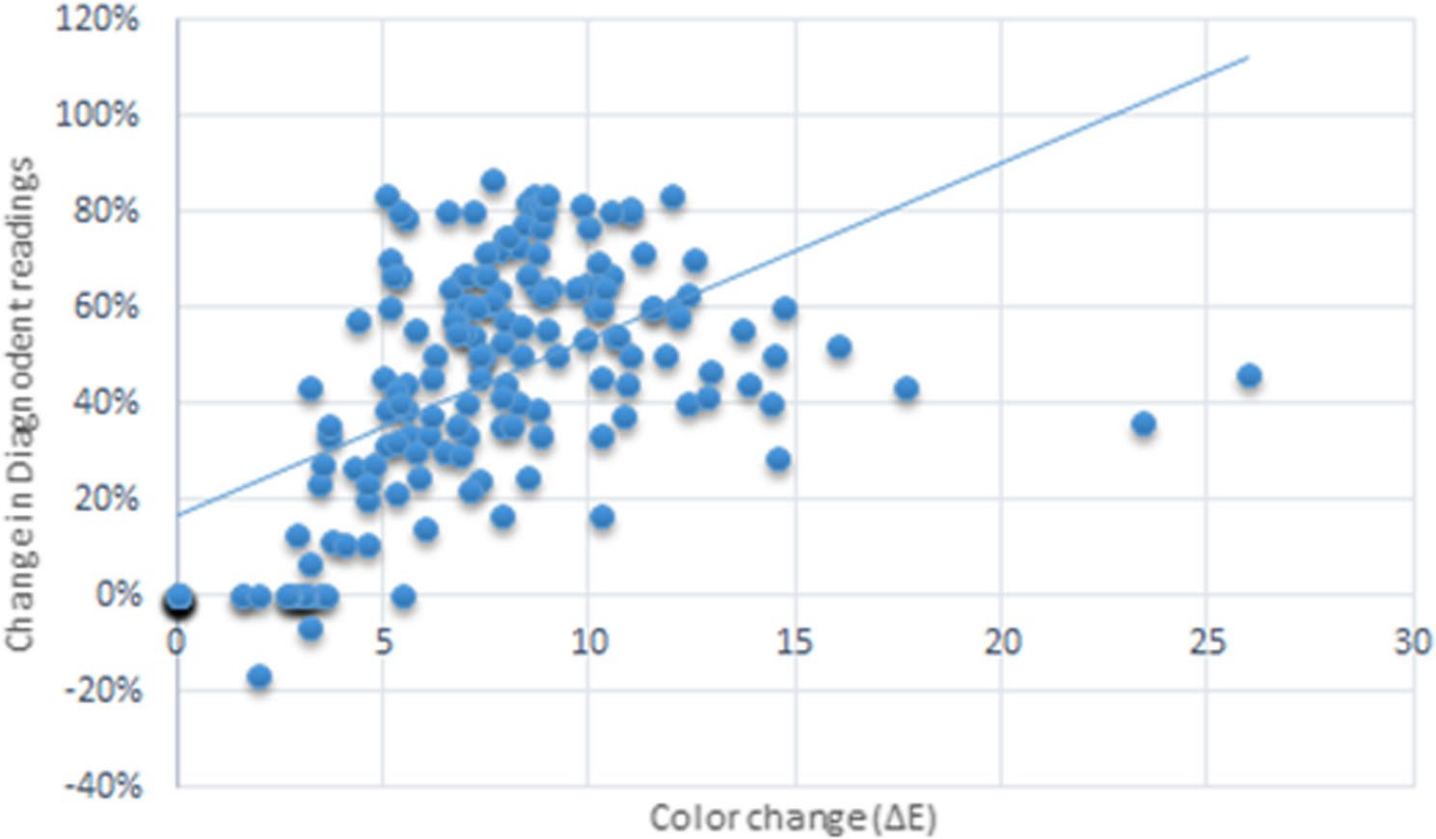
Scatter plot showing the correlation between the change in diagnodent readings and color change

As shown in Fig. 7, there was a strong positive correlation between the change in color and that in Diagnodent readings which was statistically significant [rs (95% CI) = 0.596 (0.491:0.683), *p* < 0.001].

No harms or unintended effects, such as chemical burn, taste alteration or tooth sensitivity, were recorded.

## Discussion

Dental caries is the most world wide spread chronic disease with slow progression associated with surface demineralization. In early stages, it can be detected as white spot lesion once the enamel subsurface becomes demineralized with formation of internal porosity. Such lesion becomes obvious as the refractive index of water and air inside these porosities become 1.33 and 1.0, respectively which differ than the refractive index of sound enamel that is 1.62 [30, 31].

High esthetic demand among different individuals of different ages makes the appropriate management of these lesions of a great importance. Non-invasive techniques are considered a conservative approach that aimed to conserve dental tissues. Recently, Enamel remineralization showed a great advancement either on the applied materials or their techniques of application [32].

Nowadays, NHA is strongly introduced in different fields of dentistry as a biomimetic material that permit significant tissue reconstruction. As the hydroxyapatite is the main inorganic constituent of enamel, tooth pastes containing nano-sized hydroxyapatite particles has strong affinity towards formation of tooth crystal. Moreover, the mode of action of NHA in inhibition of tooth demineralization and promotion of remineralization of WSLs lesions is now clear based upon different in vitro studies as well as clinical trials [33–35].

Xie et al., 2023 [36] in their review stated that the effectiveness of remineralization in WSLs can be boosted by using combined techniques that facilitate the management of these lesions. In addition, Deshpande et al., 2017 [32] and El Sayed et al., 2022 [37] had proved that pretreatment of demineralized enamel with mild acid had a favorable outcome on the treatment WSLs.

Microabrasion technique using Opalustre^®^ is an effective method for the removal of the superficial layer that have been pigmented following the natural remineralization by oral saliva to produce apparently normal surface. Opalustre^®^ contains hydrochloric acid with 6.6% with abrasive silicon carbide powder. Rotary application with a special cup is used to removes the superficial porous enamel layer that range between 25 and 200 μm [37].

Another more challenging step in remineralization of WSLs is to achieve good penetration towards the body of the lesion, so Justus et a, 2010 [38] and El Wazir et al. 2021 [24] had proved that using 5.25% NaOCl for enamel pretreatment is mandatory step to eliminate the organic pellicle. In addition, when NaOCl comes in contact with organic material it provides a dynamic balance due to several chemical reactions. Moreover, this reaction leads to liquefaction of organic tissues that enhance the effect of the applied remineralizing agent [24]. 

Diagnodent is a non-invasive and a reliable tool that allow early diagnosis of dental caries. It permits early detection of noncavitated. WSLs absorbers the laser light by the organic and inorganic components of the tooth that emit fluorescence in the infrared region. In case of the presence of demineralized area, the fluorescence increases and it is indicated with an audible sound. The scale ranged from 0 till 99 score. Higher sound indicates severe lesion [25, 30]. In a nut shell, the use of laser fluorescence based device is the most suitable clinical device in assessment of both demineralized and remineralized lesions by detection of the lesions^’^ severity [30, 39]. In our results interpretation, percentage of change in diagnodent reading was applied instead of the widely used difference of the absolute readings before and after different treatment protocols. As it can more precisely reflect the degree of change even in small values.

Despite the availability of different clinical trials assessed the effect of different remineralizing agents, scarce clinical studies had been conducted to investigate the efficacy of color change of any commercially available remineralizing toothpastes and their impact on the color improvement of WSLs [31, 39]. Vita Easyshade spectrophotometer was used in this clinical trial to detect color change because of its reliability, precision, and strong data consistency [40–42]. Basson et al. [43], in their review article stated that the high accuracy of Vita Easyshade could be due to the numerical measurements and the quantification of colors in a three-dimensional color space. In our results interpretation, CIEDE 2000 color difference formula (Δ*E*00) was applied because it was able to create a single-number calculation for recording small color discrepancies being more sensitive than CIE l * a * b formula.

This clinical study was carried out to assess the effect of NHA paste after different pretreatment protocols; microabrasion and NaoOCl, in comparison to NHA alone without pretreatment of WSLs in terms of enamel remineralization and color change.

Based upon our results of this clinical study, the null hypothesis was rejected as both pretreatment protocols, microabrasion and NaOCl, could improve tooth remineralization as well as color change of WSLs.

Regarding the remineralization ability of WSLs in different groups; our results revealed that group I and II that were subjected to pretreatment protocols either microabrasion or NaOCl respectively could successfully remineralize these lesions immediately while such results didn’t differ from group III in which NHA was applied directly without surface pretreatment. Whereas the negative control group did not show any difference. Concerning the remineralization ability in different intervention groups, group I, II, and III, there was constant and continuous remineralization ability by repeatable application of different corresponding remineralization protocol at 1, 2 and 6-week recall visits where the percentage of change in diagnodent reading was increasing by time indicating high remineralization power with the repeated treatment.

As mentioned before there was very few researches studied the effect of NHA either alone or after pretreatment protocol to evaluate its remineralization potential using diagnodent. In consistent with our results, Ebrahimi et al. 2017 [44], had proved that NHA toothpaste could effectively reduce the WLs and increasing the mineral content after 2 week of application while using VistaCam device. Moreover, Badr and Ragab, 2018 [45] had proved that NHA paste demonstrated better remineralization stability over the 6-month follow-up in comparison to other remineralizing agents while using diagnodent as a recording device.

A systematic review by Limeback 2023 [46] had concluded that using NHA paste in a regular base could effectively remineralize WSLs, and they had recommended performing more clinical trials to support the preliminary in vivo evidence.

It is worth mentioning that different methodology and testing device might attribute to difficulty in results interpretation.

In terms of the applied pretreatment protocols, both microabrasion and NaOCl, could successfully improve the remeneralization ability of WSLs after application of NHA toothpaste. Based upon the clinical trial carried by Deshpande et al.,2017 [32], management of WSLs could be achieved by combined treatment of microabrasion followed by remineralization with an effective and long lasting effect. While the application of NaOCL as a pretreatment protocol before NHA toothpaste showed to be an effective method that improves the remineralization capacity of NHA toothpaste. In agreement of our results, El Wazir et al.,2021 [24] had concluded that pretreatment of NaOCl could significant improve the remineralization capacity of self-assembling peptide enhancing better re-hardening action.

Regarding the color change, Muntean et al.,2019 [47] had clarified that when ΔE was greater than 3.3 this indicated an obvious visible color change that could be detected for the human eye.

Concerning the immediate effect of different treatment protocol, the Δ*E* showed a highest difference in group II where pretreatment with NaOCL was followed with NHA and group III where NHA was applied alone, as both of them could have the best optical masking of WSLs. While group I which was pretreated with microabrasion attained a good esthetic appearance of WSLs better than the negative control group. Unfortunately, there was no available research discussed the effect of color change after NaOCL pretreatment before remineralization. Manchery et al.,2019 [33] showed that NHA dentifrice could attain better results in the management of early carious lesions when evaluated with Polarizing light microscopy. Moreover, different studies evaluated the color change of WSLs after application of NHA pastes, where all of them had proved that NHA pastes could successfully mask WSLs in addition to their remineralizing effect [33, 48–50].

On the other hand, Novozhilova et al., 2025 [51] had concluded that microabrasion technique showed better masking effect for WSLs and higher color stability compared NHA. Moreover, Bhandari et al., 2019 [52] and Vats et al.,2023 [53] proved in their clinical study that the combined treatment of microabrasion-remineralization could be an efficient method for the treatment of WSLs.

After one-week, the difference of color change Δ*E* was obvious where group II which was pretreated with NaOCL followed with NHA showed the highest degree of color change than group III and other groups. Also pretreatment with microabrasion before application of NHA paste in group II seemed to have a satisfying esthetic masking of WSLs. It was previously mentioned that scare studies evaluated the color change using Vita easy shade of WSLs after different pretreatment protocol before application NHA paste.

Our results revealed that repeated application after two and six weeks of different protocols could successfully mask the WSLs where Δ*E* showed a great difference in all groups in comparison to the negative control group. This was in accordance with Heravi et al., 2018 [48] and Eliwa et al., 2022 [54] that concluded that continuous application of NHA paste could improve the color appearance of WSLs.

An important note had been recorded in our study, as the best results of the Diagodent were recorded at week 6, while for Vita Easy Shade, at week 1. This might be attributed to the fact that tooth remineralization effect could last for a longer time as the minerals could strongly invade the depth of white spot lesion as well as good oral hygiene measures could improve the remineralizing effect which was reflected by the better results of Diagnodent after 6 weeks follow up. However, the optical effect recorded with Vitaeasy shade after tooth remineralization could be easily affected by other factors like type of diet and frequency of tooth brushing which could end up with better immediate esthetic outcome than that after 6 weeks follow up.

Finally, our results showed a strong correlation between remineralization capacity and color improvement of WSLs. As repeated application of different intervention protocols, could enhance tooth remineralization as well as improve the optical appearance of WSLs.

The main limitation of this study is the short follow up period; otherwise an extended time frame might predict the longevity of various treatment options. Also, the effects of diet and frequency of tooth brushing as confounding factors were not correlated with the obtained results. Split mouth design may standardize many factors but it wasn’t available in our subjects. Also total blinding to remineralizing materials were not possible due to visual difference between pastes and gel.

On the basis of the results of this study, we recommend performing further high quality RCTs with different remineralizing materials pretreated by various treatment modalities. Studies should be with longer follow up periods are needed. Non-fluoridated products should gain popularity in the future among dentists and patients for managing WSLs in children.

## Conclusions

NHA could be a good non fluoridated option to remineralize and improve the color of WSL at different time intervals in contrast to oral hygiene measures alone. Pretreatment with microabrasion or NaOCl before the application of NHA toothpaste could enhance the remineralization capability and improve the color of WSLs compared with its use alone after repeated application.

## Supplementary Information

Supplementary Material 1

## Data Availability

The datasets used and/or analyzed during the current study are available from the corresponding author upon reasonable request. All efforts were made to avoid compromising an individual’s privacy.

## Abbreviations

WSL: White Spot Lesion
NHA: Nanohydroxyapatite
NaOCl: Sodium hypochlorite
CPP-ACP: Casein Phosphopeptide Amorphous Calcium Phosphate
HA: Hydroxyapatite
RCT: randomized controlled trial
CONSORT: Consolidated Standards of Reporting Trials
CIELAB: Commission Internationale de l’Eclairage L* a* and b*, CI: Confidence Intervals
IQR: Interquartile Range
FDR: False Discovery Rate
Micro Ab: Microabrasion

## References

[1] Blanchet I, Camoin A, Tardieu C, Jacquot B. Microabrasion in the management of enamel discolorations in pediatric dentistry”. J Clin Pediatr Dent. 2023;47:17–26. 10.22514/jocpd.2022.015. 36627216 10.22514/jocpd.2022.015

[2] Bailey DL, Adams GG, Tsao CE, Hyslop A, Escobar K, Manton DJ, et al. Regression of post-orthodontic lesions by a remineralizing cream. J Dent Res. 2009;88:1148–53. 10.1177/0022034509347168. 19887683 10.1177/0022034509347168

[3] Petersen PE, Ogawa H. Prevention of dental caries through the use of fluoride – The WHO approach. Community Dent Health. 2016;33:66 – 8 PMID: 27352461. 27352461

[4] Kanduti D, Sterbenk P, Artnik B. Fluoride: a review of use and effects on health. Mater Sociomed. 2016;28:133–7. 10.5455/msm.2016.28.133-137. 27147921 10.5455/msm.2016.28.133-137 PMC4851520

[5] Elsayed HH. Evaluation of the state and shade of white spot lesions after treatment with different remineralizing agents (an in-vivo comparative study). Al-Azhar J Dent Sci. 2021;24(3):239–45. 10.21608/ajdsm.2020.46840.1123.

[6] Melo MA, Guedes SF, Xu HH, Rodrigues LK. Nanotechnology-based restorative materials for dental caries management. Trends Biotechnol. 2013;31(8):459–67. 10.1016/j.tibtech.2013.05.010. 23810638 10.1016/j.tibtech.2013.05.010 PMC3845439

[7] Sadat-Shojai M, Khorasani MT, Dinpanah-Khoshdargi E, Jamshidi A. Synthesis methods for nanosized hydroxyapatite with diverse structures. Acta Biomater. 2013;9(8):7591–621. 10.1016/j.actbio.2013.04.012. 23583646 10.1016/j.actbio.2013.04.012

[8] -Pagano S, Coniglio M, Valenti C, et al. Biological effects of resin monomers on oral cell populations: descriptive analysis of literature. Eur J Paediatr Dent. 2019;20(3):224–32. 10.23804/ejpd.2019.20.03.11. PMID: 31489823. 31489823 10.23804/ejpd.2019.20.03.11

[9] Ebadifar A, Nomani M, Fatemi SA. Effect of nano-hydroxyapatite toothpaste on microhardness of artificial carious lesions created on extracted teeth. J Dent Res Dent Clin Dent Prospects. 2017;11(1):14–7. 10.15171/joddd.2017.003. 28413590 10.15171/joddd.2017.003 PMC5390120

[10] Robertson MA, et al. Mi paste plus to prevent demineralization in orthodontic patients: a prospective randomized controlled trial. Am J Orthod Dentofacial Orthop. 2011;140(5):660–8. 10.1016/j.ajodo.2010.10.025. 22051486 10.1016/j.ajodo.2010.10.025

[11] Sundfeld RH, Franco LM, Gonçalves RS, de Alexandre RS, Machado LS, Neto DS. <article-title update="added">Accomplishing esthetics using enamel microabrasion and bleaching—a case report. Oper Dent. 2014;39:223–7. 10.2341/13-002-S. 23919624 10.2341/13-002-S

[12] Guerrieri A, Gaucher C, Bonte E, Lasfargues J. Detection and diagnosis of initial caries lesions. Br Dent J. 2012;213(11):551–7. 10.1038/sj.bdj.2012.1087. 23222326 10.1038/sj.bdj.2012.1087

[13] Jayarajan J, Janardhanam P, Jayakumar P. Efficacy of CPP-ACP and CPP-ACPF on enamel remineralization – An in vitro study using scanning electron microscope and DIAGNOdent^®^. Indian J Dent Res. 2011;22(77). 10.4103/0970-9290.80001. PMID: 21525682. 10.4103/0970-9290.80001 21525682

[14] -Paul SJ, Peter A, Rodoni L, Pietrobon N. Conventional visual vs spectrophotometric shade taking for porcelain-fused-to-metal crowns: a clinical comparison. Int J Periodont Rest Dent. 2004;24:222 – 31 PMID: 15227770. 15227770

[15] Alghazali N, Burnside G, Smith PW, Preston AJ, Jarad FD. Performance assessment of vita easy shade spectrophotometer on colour measurement of aesthetic dental materials. Eur J Prosthodont Restor Dent. 2011;19(4):168. PMID: 22645803. 22645803

[16] -Schulz KF, Altman DG, Moher D. CONSORT 2010 statement: updated guidelines for reporting parallel group randomized trials. Trials. 2010;11(1):1–8. PMID: 21686296, PMCID: PMC3116666. 21686296 PMC3116666

[17] World Medical Association Declaration of Helsinki: ethical principles for medical research involving human subjects. JAMA. 2013;310(20):2191–4. 10.1001/jama.2013.281053. 10.1001/jama.2013.281053 24141714

[18] Mohammed HH, Mohamed MS, Elsamolly WM. Evaluation of the state and shade of white spot lesions after treatment with different remineralizing agents (an in-vivo comparative study). Al-Azhar JDS. 2021;24(3):239–45. 10.21608/ajdsm.2020.46840.1123.

[19] Uttley J. Power Analysis, Sample Size, and Assessment of Statistical Assumptions—Improving the Evidential Value of Lighting Research. LEUKOS. 2019;15(2–3):143–62. 10.1080/15502724.2018.1533851.

[20] Ando M, Shaikh S, Eckert G. Determination of caries lesion activity: reflection and roughness for characterization of caries progression. Oper Dent. 2018;43(3):301–6. 10.2341/16-236-L. 29676973 10.2341/16-236-L

[21] Cazzolla AP, De Franco AR, Lacaita M, Lacarbonara V. Efficacy of 4-year treatment of Icon infiltration resin on postorthodontic white spot lesions. BMJ Case Rep. 2018. 10.1136/bcr-2018-225639. 30021744 10.1136/bcr-2018-225639 PMC6058148

[22] AlFeel J, et al. Evaluating the effect of Clinpro Tooth Crème on remineralization of pre-carious white spot lesions in anterior primary teeth: randomized controlled clinical trial. Pediatr Dent J. 2021. 10.1016/j.pdj.2021.03.001.

[23] -Hussain MAA, Rafeeq RA. Efficacy of three commercially available fluoride releasing varnishes in remineralization of artificial white spot lesions evaluated by laser fluorescence: an in vitro study. Dent Hypotheses. 2022;13:117–20. 10.4103/denthyp.denthyp_79_22.

[24] Elwazir AA, Fahmy OM, Nabih SM. Effect of sodium hypochlorite (NaOCl) for pretreatment of early demineralized enamel lesions in enhancing the remineralization capacity of self-assembling peptide (in-vitro study). J Fundam Clin Res. 2021;1(1):1–16. 10.21608/jfcr.2021.181031.

[25] Gençer MDG, Kirzioğlu Z. A comparison of the effectiveness of resin infiltration and microabrasion treatments applied to developmental enamel defects in color masking. Dent Mater J. 2019;38(2):295–302. 10.4012/dmj.2018-074. 30713284 10.4012/dmj.2018-074

[26] -ElAziz RHA, Gadallah LK, Saleh RS. Evaluation of charcoal and sea salt–lemon-based whitening toothpastes on color change and surface roughness of stained teeth. J Contemp Dent Pract. 2022;23(2):169–75. PMID: 35748445. 35748445

[27] Casado BGS, Moraes SLD, Souza GFM, et al. Efficacy of dental bleaching with whitening dentifrices: A systematic review. Int J Dent. 2018;2018:7868531. 10.1155/2018/7868531. 30510576 10.1155/2018/7868531 PMC6232812

[28] Tomczak M, Tomczak E. The need to report effect size estimates revisited. An overview of some recommended measures of effect size. Trends Sport Sci. 2014;21(1):19–25.

[29] Core Team -R. R: A language and environment for statistical computing. R Foundation for Statistical Computing, Vienna, Austria. 2024. Available from: https://www.R-project.org/

[30] Dhanya K, Chandra P, Anandakrishna L, Karuveettil V. A comparison of NovaMin™ and casein phosphopeptide amorphous calcium phosphate fluoride on enamel remineralization – an *in vitro* study using scanning electron microscope and DIAGNOdent®. Contemp Clin Dent. 2021;12:301–7. 10.4103/ccd.ccd_240_19. 34759689 10.4103/ccd.ccd_240_19 PMC8525807

[31] Shah Y, Deshpande A, Jain A, Jaiswal V, Andharia M. Effectiveness of resin infiltration (ICON) and microabrasion remineralization technique with two remineralizing agents (Tooth Mousse and Toothmin) on permanent incisor hypoplasia – a randomized clinical trial. J Indian Soc Pedod Prev Dent. 2023;41(3):204–15. 10.4103/jisppd.jisppd_245_23. 37861634 10.4103/jisppd.jisppd_245_23

[32] Deshpande AN, Joshi NH, Pradhan NR, Raol RY. Microabrasion-remineralization: an innovative approach for dental fluorosis. J Indian Soc Pedod Prev Dent. 2017;35:384–8. 10.4103/JISPPD.JISPPD_216_16. 28914255 10.4103/JISPPD.JISPPD_216_16

[33] -Manchery N, John J, Nagappan N, Subbiah GK, Premnath P. Remineralization potential of dentifrice containing nanohydroxyapatite on artificial carious lesions of enamel: A comparative in vitro study. Dent Res J. 2019;16:310–7. PMID: 31543937, PMCID: PMC6749857. PMC6749857 31543937

[34] Bennett T, Amaechi A, Alshareif DO, Abdul Azees PA, Shehata MA, Lima PP et al. Anti-caries evaluation of a nano-hydroxyapatite dental lotion for use after toothbrushing: An in situ study. J Dent. 2021;115:103863 PMID: 34743963 10.1016/j.jdent.2021.103863 10.1016/j.jdent.2021.103863 34743963

[35] -Badiee M, Jafari N, Fatemi S, Ameli N, Kasraei S, Ebadifar A. Comparison of the effects of toothpastes containing nanohydroxyapatite and fluoride on white spot lesions in orthodontic patients: A randomized clinical trial. Dent Res J. 2020;17:354–9. PMID: 33343843. PMCID: PMC7737819. PMC7737819 33343843

[36] Xie Z, Yu L, Li S, Li J, Liu Y. Comparison of therapies of white spot lesions: A systematic review and network meta-analysis. BMC Oral Health. 2024;4. 10.1186/s12903-023-03076-x. PMID: 37264364, PMCID: PMC. 10.1186/s12903-023-03076-x PMC10233982 37264364

[37] El Sayed HH, Abo ELezz AF, Fahmy OM. Effect of three treatment modalities for white spot enamel lesions on enamel surface roughness and microhardness. Dent Sci Updates. 2022;3(2):127–37. 10.21608/dsu.2022.101372.1086.

[38] Justus R, Cubero T, Ondarza R, Morales F. A new technique with sodium hypochlorite to increase bracket shear bond strength of fluoride releasing resin-modified glass ionomer cements: comparing shear bond strength of two adhesive systems with enamel surface deproteinization before etching. Semin Orthod. 2010;16:66–75. 10.1053/j.sodo.2009.12.006.

[39] Zaazou MH, Saleh RS, Hassan SN, Abdelnabi A, Zaki ZM, Hamdy TM, et al. Effectiveness of low-viscosity resin infiltration (Icon) on color change of enamel white spot lesions: 1-year follow-up clinical study. Bull Natl Res Cent. 2024;48:62.

[40] -Candan M, Ünal M. The effect of various inhaled asthma medications on the color stability of paediatric dental restorative materials. BMC Oral Health. 2024;24(1):384. 38528493 10.1186/s12903-024-04118-8 PMC11289941

[41] Guler S, Unal M. The evaluation of color and surface roughness changes in resin based restorative materials with different contents after waiting in various liquids: an SEM and AFM study. Microsc Res Tech. 2018;81(12):1422–33. 30295386 10.1002/jemt.23104

[42] -Unal MU, Oztas NU. Remineralization capacity of three fissure sealants with and without gaseous Ozone on non-cavitated incipient pit and fissure caries. J Clin Pediatr Dentistry. 2015;39(4):364–70. 10.17796/1053-4628-39.4.364 26161609

[43] Basson RA, Grobler SR, Kotze TJ, vW et al. Guidelines for the selection of tooth whitening products amongst those available on the market. SADJ. 2013;68(3):122-9 PMID: 23951776. 23951776

[44] Ebrahimi M, Mehrabkhani M, Ahrari F, Parisay I, Jahantigh M. The effects of three remineralizing agents on regression of white spot lesions in children: a two-week, single-blind, randomized clinical trial. J Clin Exp Dent. 2017;9(5):e641-8. 10.4317/jced.53582. 28512540 10.4317/jced.53582 PMC5429475

[45] Badr S, Ragab H. The effectiveness of a nano-hydroxyapatite paste and a tri-calcium phosphate fluoride varnish in white spot lesions remineralization (randomized clinical trial). EDJ. 2018;64(3):95–103. 10.21608/edj.2018.77273.

[46] Limeback H, Meyer F, Enax J. Tooth whitening with hydroxyapatite: a systematic review. Dent J. 2023;11:50. 10.3390/dj11020050. 10.3390/dj11020050 PMC9955010 36826195

[47] Muntean A, Sava S, Delean AG, Mihailescu AM, Dumitrescu LS, Moldovan M, et al. Toothpaste composition effect on enamel chromatic and morphological characteristics: in vitro analysis. Materials. 2019;12:2610. 10.3390/ma12162610. 31426296 10.3390/ma12162610 PMC6720655

[48] -Heravi F, Ahrari F, Tanbakuchi B. Effectiveness of MI paste plus and remin pro on remineralization and color improvement of postorthodontic white spot lesions. Dent Res J. 2018;15:95–103. PMID: 29576772, PMCID: PMC5858078. PMC5858078 29576772

[49] da Brezolini Freiria AC, Guanipa Ortiz MI, de Souza Sobral DF, Aguiar FHB, Nunes Leite Lima DA. Nano-hydroxyapatite-induced remineralization of artificial white spot lesions after bleaching treatment with 10% carbamide peroxide. J Dent. 2022;115:103863. 10.1111/jerd.12969. 10.1111/jerd.12969 36205242

[50] Juntavee A, Juntavee N, Hirunmoon P. Remineralization potential of nanohydroxyapatite toothpaste compared with tricalcium phosphate and fluoride toothpaste on artificial carious lesions. Int J Dent. 2021;2021:1–9. 10.1155/2021/5588832. 10.1155/2021/5588832 PMC8007336 33824661

[51] Novozhilova N, Mun A, Polyakova M, Mikheikina A, Zaytsev A, Babina K. Color change and color stability of white spot lesions treated with resin infiltration, microabrasion, or nano-hydroxyapatite remineralization: an in vitro study. Dent J. 2025;13:112. 10.3390/dj13030112. 10.3390/dj13030112 PMC11940927 40136740

[52] Bhandari R, Thakur S, Singhal P, Chauhan D, Jayam C, Jain T. In vivo comparative evaluation of esthetics after microabrasion and microabrasion followed by casein phosphopeptide–amorphous calcium fluoride phosphate on molar incisor hypomineralization affected incisors. Contemp Clin Dent. 2019;10:9–15. 10.4103/ccd.ccd_852_17. 32015635 10.4103/ccd.ccd_852_17 PMC6975009

[53] Vats S, Sinha DJ, Sharma N, Meena A. Management of enamel stains using a combination technique of microabrasion and remineralizing agent. Ann Dent Spec. 2023;2023:1–5.

[54] Eliwa ME, Aidaros N, Kamh R. A comparative evaluation of remineralization potential of nano-seashell, nano-pearl, and nano-hydroxyapatite pastes versus fluoride-based toothpaste on non-cavitated initial enamel lesion: an in vitro study. Egypt Dent J. 2022;68:1025–41. 10.21608/EDJ.2022.102806.1840.

